# Overview of MMP-13 as a Promising Target for the Treatment of Osteoarthritis

**DOI:** 10.3390/ijms22041742

**Published:** 2021-02-09

**Authors:** Qichan Hu, Melanie Ecker

**Affiliations:** Department of Biomedical Engineering, University of North Texas, Denton, TX 76203, USA; QichanHu@my.unt.edu

**Keywords:** osteoarthritis, cartilage, type II collagen, matrix metalloproteinases, MMP-13, regulation, inhibitor

## Abstract

Osteoarthritis (OA) is a common degenerative disease characterized by the destruction of articular cartilage and chronic inflammation of surrounding tissues. Matrix metalloproteinase-13 (MMP-13) is the primary MMP involved in cartilage degradation through its particular ability to cleave type II collagen. Hence, it is an attractive target for the treatment of OA. However, the detailed molecular mechanisms of OA initiation and progression remain elusive, and, currently, there are no interventions available to restore degraded cartilage. This review fully illustrates the involvement of MMP-13 in the initiation and progression of OA through the regulation of MMP-13 activity at the molecular and epigenetic levels, as well as the strategies that have been employed against MMP-13. The aim of this review is to identify MMP-13 as an attractive target for inhibitor development in the treatment of OA.

## 1. Introduction

Osteoarthritis (OA) is one of the most common degenerative joint diseases primarily among the elderly who exhibit typical clinical symptoms such as joint pain, swelling, stiffness, and restricted movement. This may lead to decreased productivity and quality of life among the patients, in addition to an increased socioeconomic burden to the patients and the society as a whole [1,2]. According to the statistics from 2017, over 303 million people worldwide suffer from OA, which makes this disease a non-negligible subject [3].

**Figure 1:**
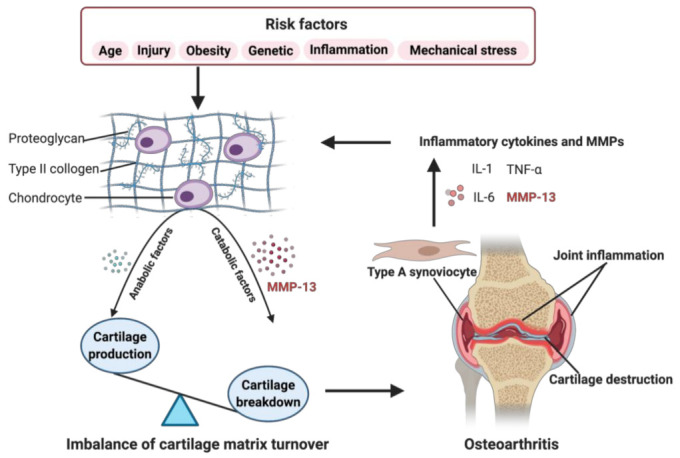
Role of matrix metalloproteinase-13 (MMP-13) in osteoarthritis (OA) pathogenesis. When some OA risk factors lead to increased expression of chondrocytes’ catabolic factors, like MMP-13, the balance tips toward a net loss of cartilage. MMP-13 is the primary catabolic factor involved in cartilage degradation through its particular ability to cleave type II collagen. The breakdown products of cartilage stimulate the type A synoviocytes to release inflammatory cytokines and MMPs, like tumor necrosis factor alpha (TNF-α), interleukin (IL)-1, IL-6, and MMP-13, which, in turn, enhance a more comparable catabolic effect on chondrocyte metabolism, accelerating the progression of OA. Created with BioRender.com.

The specific cause of OA remains elusive. Still, multiple risk factors contribute to the development of OA, including traumatic knee injury, obesity, genetic predisposition, abnormal mechanical stress, and other inflammation caused by infection or surgery, in addition to aging (Figure 1) [4,5,6]. Recent research has indicated that OA affects the joints’ entire structures, including articular cartilage, subchondral bone, synovial membrane, intra-articular fat pads and intraarticular supporting fibrocartilaginous structures (e.g., menisci), particularly those in the knees, hands, and hips [7,8,9,10]. The common structural characteristics of OA are chronic inflammation, progressive destruction of articular cartilage, and subchondral bone sclerosis, especially, the irreversible degradation of articular cartilage is central in the pathological process of OA [11].

Articular cartilage is a thin layer of connective tissue composed of chondrocytes and extracellular matrix (ECM) without blood vessels. It has a four-layered structure, including the superficial, middle, deep, and calcified cartilage zones, with a sparse distribution of chondrocytes in the ECM of various zones [12]. The ECM is primarily composed of proteoglycans and collagens, and other less-abundant components, such as elastin, gelatin, and matrix glycoproteins [13]. Type II collagen is the major structural protein of cartilage, forming a network structure of ECM with aggrecan and other proteoglycans tangled within it [14]. The regular turnover of these matrix components is very slow and mediated by the chondrocytes, which synthesize these components and the proteolytic enzymes responsible for their breakdown [15]. The balance between anabolism and catabolism in articular cartilage is regulated by a complex network of factors, but it is mainly maintained by MMPs and its endogenous tissue inhibitors of metalloproteinases (TIMPs) [16]. MMP-13 (collagenase 3) is the key enzyme in the cleavage of type II collagen and plays a pivotal role in the breakdown of cartilage in osteoarthritic joints [17].

As shown in Figure 1, risk factors may cause an increased expression of both, anabolic and catabolic factors. However, the catabolic factors increase much more than anabolic factors, causing a disbalance. For example, the chondrocytes secret more MMP-13, resulting in enhanced degradation of ECM, leading to the balance tips toward a net loss of cartilage [18]. The breakdown products of cartilage are released into the synovial fluid and phagocytized by resident macrophages, such as type A synoviocytes containing vacuoles related to phagocytic function [17,19,20]. When the production of these decomposing particles exceeds the system’s ability to eliminate them, they become mediators of inflammation. The exposition of digested material through the major histocompatibility complex class I and class II make the type A synoviocytes dialogue with the lymphocytes through their T cell receptors. The invading T cells in the synovial cavity stimulate type A synoviocytes into an inflammatory state, producing various inflammatory cytokines and MMPs, like TNF-α, IL-1, IL-6, and MMP-13, which, in turn, enhance a more comparable catabolic effect on chondrocyte metabolism, accelerating the progression of OA [20]. Several signaling pathways are involved in regulating catabolic events in OA, including nuclear factor kappa-light-chain-enhancer of activated B cells (NF-κB), phosphoinositide 3-kinase/protein kinase B (PI3K/AKT), mitogen-activated protein kinase (MAPK), and others, which modulate the expression of cytokines, chemokines, and matrix-degrading enzymes [21].

Currently, there is no effective treatment to reverse the destructive process of articular cartilage. Thus, the treatment is limited to symptom-relieving approaches involving medications, physical and occupational therapy, and surgical procedures [22]. These treatments aim to relieve pain, maintain joint flexibility, improve joint function and quality of life, and to slow down the disease’s progression. However, there are many side effects associated with these conventional approaches. For example, the damage to liver, kidney, and cardiovascular system with long-term use of acetaminophen and non-steroidal anti-inflammatory drugs (NSAIDs) [23], and the risk of reoperation for infectious complications after arthroplasty [24]. Other novel treatments have also been extensively studied, like low-dose radiation [25] and intra-articular injection, including agonist for the transient receptor potential cation channel subfamily V member 1 (e.g., Capsaicin) [26], IL-1α/β dual variable domain immunoglobulin (e.g., Lutikizumab) [27], a humanized monoclonal antibody (e.g., Galcanezumab) [28], and regenerative medicine (e.g., platelet-rich plasma or mesenchymal stem cell) [29,30]. However, these treatments are limited to clinical trials with no or inadequate efficacy.

To overcome current limitations and improve patient outcome, there is an urgent requirement to develop effective therapies that have fewer side effects for OA. A large body of studies revolved around generating and evaluating chemical inhibitors of MMPs, which have shown to inhibit the destruction of cartilage in some animal models of OA [31]. However, owing to the high degree of structural similarity across their active sites, many MMP inhibitors have failed in clinical trials due to low selectivity and side effects [32]. Given this, pharmaceutical research has mainly focused on discovering potent inhibitors of MMP-13 displaying a high degree of selectivity over other MMPs [33].

In this review, the databases used were PubMed and Google Scholar with appropriate keywords (osteoarthritis, pathogenesis, cartilage degradation, MMP-13, epigenetic regulation, and synthetic inhibitor). Overall, approximately 2000 references were initially identified from 2000 until 2020. After the initial screening of titles and abstracts, the articles without mentioning MMP-13 were excluded. We analyzed the included literature to get a comprehensive overview of OA pathogenesis and possible biomarkers and target molecules for OA treatment. Subsequently, determine the topic and component issues. Additional information retrieval was also made when it comes to specific problems.

## 2. Basic Aspects of MMP-13

### 2.1. Structure

**Figure 2:**
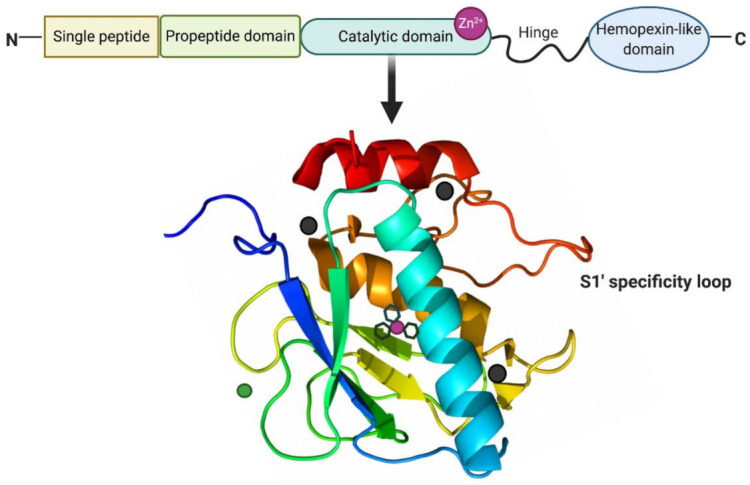
Structure of MMP-13. MMP-13 typically consists of a highly conserved signal peptide, a propeptide domain, a catalytic domain, a proline-rich hinge region, and a C-terminal hemopexin-like domain. The catalytic domain of MMP-13 is represented by the crystal structure. The structural zinc ion is in green, the catalytic zinc ion is in magenta, and three calcium ions are in dark grey. Three histidine residues in black sticks coordinate the catalytic zinc ion. The highly flexible S1′ specificity loop as part of hydrophobic S1′ pocket is a determining factor for the selective inhibitors of MMP-13. Created with BioRender.com.

MMPs are a family of zinc-dependent proteolytic enzymes responsible for the cleavage of a variety of ECM proteins [34]. They are mainly classified into collagenases (MMP-1, -8, -13, and -18), gelatinases (MMP-2 and -9), membrane-type MMPs (MMP-14, -15, -16, -17, -24, and -25) and others (MMP-7, -12, -19, -20, -23, -26, and -28) [35]. MMPs are multi-domain proteins with a highly conserved signal peptide, a propeptide domain, and a catalytic domain. Except for MMP-7, -23, and -26, all MMPs also contain a proline-rich hinge region and a C-terminal hemopexin-like domain [36]. As shown in Figure 2, the spherical catalytic domains share the same structural organization: three α-helixes, five β-sheets, connected by eight loops. Additionally, they contain a catalytic zinc ion coordinated by three histidine residues, a structural zinc ion, and three structural calcium ions required for enzyme stability [37]. The specificity of the MMP-substrate interaction depends on specific subsites or pockets (S) within the MMP molecule that interacts with corresponding substituents (P) in the substrate. The pockets localized on both sides of the catalytic zinc ion (Left: S1, S2, S3, ... Sn; Right: S1′, S2′, S3′, ... Sn’ ) confer binding specificity to the substrate P1, P2, P3, … Pn and primed P1′, P2′, P3′, ... Pn’ substituents, respectively [38]. Of these pockets, the S1′ is the most variable in both the amino acid makeup and depth of the pocket [39]. The S1′ pocket may be shallow (e.g., MMP-1 and MMP-7), intermediate (e.g., MMP-2, MMP-8 and MMP-9), or deep (e.g., MMP-3, MMP-11, MMP-12, MMP-13 and MMP-14) [40,41]. The large hydrophobic S1′ pocket of MMP-13 has a highly flexible “S1′ specificity loop (Ω-loop)” consisting of residues 245–253, which has been suggested to be a determining factor for the selective binding of inhibitors of MMP-13 [42].

### 2.2. Zymogen Activation

MMPs are produced by various tissues and cells [43]. They are synthesized as inactive zymogens (pro-MMPs), and this inactive form is maintained by a “cysteine switch” motif PRCGXPD in which the cysteine residue coordinates with the Zn^2+^ in the catalytic domain [44]. Proteolytical activation of all pro-MMPs often takes place extracellularly through cleavage of their pro-domains by other MMPs and protease [45]. MMP-13 is produced as a 60 kDa precursor form (proMMP-13), which can be activated by MT1-MMP on the cell surface, more efficient in the presence of active MMP-2 [46]. Additionally, plasmin has been shown to activate proMMP-13 with the involvement of the urokinase-type plasminogen activator-plasmin cascade [47].

### 2.3. Role in OA

Most MMPs, including MMP-1, MMP-2, MMP-3, MMP-8, MMP-9, MMP-10, MMP-13, and MMP-14, are involved in the turnover of ECM and the associated destruction of articular cartilage in OA [48]. Still, the soluble collagenases, MMP-1, MMP-8, and MMP-13, are crucial for this destruction to occur, especially MMP-13 predominates [48]. The preferred substrate for MMP-13 is type II collagen, which is cleaved five times faster than collagen I, six times faster than collagen III, and more readily than by other collagenases [49]. Again, the importance of MMP-13 in type II collagen cleavage is supported by the destabilization of the medial meniscus (DMM) model of OA when performed in MMP-13^−/−^ mice. In this model, MMP-13^−/−^ mice showed less tibial cartilage erosion than wild-type mice at 8 weeks post-surgery [50]. Conversely, cartilage-restricted expression of a constitutively active MMP-13 in mice resulted in joint pathology of the kind observed in OA [51]. Thus, MMP-13 is particularly related to the degradation of articular cartilage in OA by aggressively breakdown of type II collagen. Though, as is mentioned, it is involved principally in the degradation of type II collagen, MMP-13 also targets other matrix molecules such as type I, III, IV, IX, X collagen, perlecan, osteonectin, and proteoglycan [52], and it is likely involved in matrix turnover in healthy cartilage.

## 3. Molecular Regulation of MMP-13

**Figure 3:**
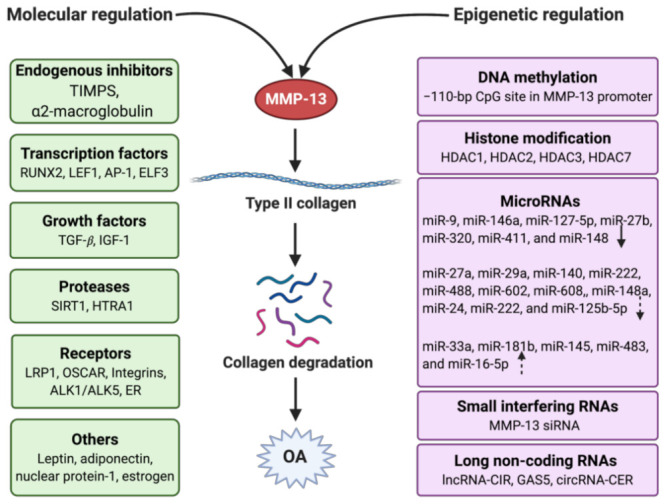
Regulation of MMP-13 in OA with the molecular and epigenetic mechanism. Molecular regulation involves endogenous inhibitors, transcription factors, growth factors, proteases, receptors, and other mediators. Epigenetic regulation contains DNA methylation, histone modification, and non-coding RNAs, which include microRNAs, small interfering RNAs, and long non-coding RNAs. The arrows in the microRNAs frame mean the miRNAs have different types of regulation (solid down-arrow: direct downregulation, dotted down-arrow: indirect downregulation, dotted up-arrow: indirect upregulation). Created with BioRender.com.

MMP-13 is a well-known key player in the pathology of early OA due to its capacity to directly or indirectly initiate the degradation of a wide range of downstream matrix and collagen components via its regulatory factors through specific signaling pathways [53]. These factors include endogenous inhibitors, transcriptional factors, promoters, growth factors, receptors, proteases, hormones, and others (Figure 3). They work together in the integrated network to regulate the activity of MMP-13 by triggering specific pathways.

### 3.1. Endogenous Inhibitors

In normal physiology, MMPs are required for various processes such as tissue remodeling, embryonic development, angiogenesis, cell adhesion, and wound healing. Alterations in specific MMPs could lead to various pathological disorders. Therefore, MMP activity is constitutively regulated by endogenous inhibitors, such as tissue inhibitors of metalloproteinases (TIMPs) and α2-macroglobulin [54].

TIMPs (TIMP-1, TIMP-2, TIMP-3, and TIMP-4) are specific inhibitors that bind MMPs in a 1:1 stoichiometry. The overall shape of TIMP molecule is like a wedge, which slots into the active-site cleft of an MMP like that of the substrate. TIMPs inhibit all MMPs tested so far, except that TIMP-1 fails to inhibit MT1-MMP, MT2-MMP, MT3-MMP, and MT5-MMP [55]. They can hinder both activated MMPs and the conversion of pro-MMPs to activated MMPs, as well as regulate a variety of other cellular functions which may or may not directly involve MMPs [56]. TIMP-3, in particular, has been ascribed a chondroprotective role in cartilage, which was demonstrated by TIMP-3^−/−^ mice exhibiting increased cartilage collagen destruction [57]. The addition of exogenous TIMP-3 by intraarticular injection blocks cartilage breakdown in a rat meniscal tear model of OA mainly due to the potency against MMP-13 [58]. Although TIMP-3 is elevated in the cartilage of OA patients compared with normal cartilage, MMP-13 is increased more significantly than TIMP-3 [59]. Therefore, TIMP-3 is insufficient to regulate MMP-13 activity, resulting in the progression of OA.

In addition to endogenous TIMPs, α2-macroglobulin is another endogenous MMP inhibitor found in blood and tissue fluids. MMP activity is partly regulated by α2-macroglobulin and related proteins. Human α2-macroglobulin is a glycoprotein consisting of four identical subunits. α2-macroglobulin is a wide-spectrum proteinase inhibitor that inhibits most endopeptidases, including MMPs, by entrapping them within the macroglobulin. The complex is then rapidly internalized and cleared by endocytosis via low-density lipoprotein receptor-related protein-1 [60].

### 3.2. Transcription Factors

Runt-related transcription factor 2 (Runx2) is a crucial transcription factor associated with OA development [61]. Hirata et al. elucidated its molecular mechanism underlying the endochondral ossification during OA development with the compound knockout of C/EBPb (CCAAT/enhancer-binding protein-b) and Runx2 in mice, showing C/EBPb and RUNX2, with MMP-13 as the target and HIF-2α as the inducer, control cartilage degradation [62]. Lymphoid enhancer-binding factor 1 (LEF1) is a transcription factor primarily involved in the canonical Wnt/β-catenin signaling pathway to regulate MMP-13 expression in chondrocytes induced by IL-1 β [63]. It is accomplished by transactivating MMP-13 promoter activity through LEF1/β-catenin binding to the LEF1 binding site in the 3′ region of the MMP-13 genomic locus. Moreover, the same group in another study showed that IL-1β stimulation increases physical interactions between the 3′ region-bound LEF1 and promoter-bound transcription factors AP-1 or NF-kB, leading to synergistic upregulation of MMP-13 gene expression [64]. The E-74 like factor 3 (ELF3) is a transcription factor induced by proinflammatory factors in various cell types, including OA cartilage and synovium [65]. ELF3 directly controls MMP-13 promoter activity by targeting an E26 transformation-specific binding site enhanced by IL-1β stimulation in chondrocytes. Consistently, the IL-1β-induced MMP-13 expression is inhibited in primary human chondrocytes by siRNA-ELF3 knockdown and in chondrocytes from ELF3^−/−^ mice, indicating that ELF3 as a procatabolic factor contributes to cartilage remodeling and degradation by regulating MMP-13 gene transcription [66]. Hairy and enhancer of split-1 (Hes1) is a transcription factor that is activated by Notch signaling. Sugita et al. reported that Hes1 is involved in the upregulation of expression of MMP-13 [67]. Hes1-knockout mice exhibited the suppression of cartilage destruction and decreased MMP-13 expression in a surgically induced OA model, suggesting that Hes1 acts through Notch-Hes1 signaling [67].

### 3.3. Growth Factors

Transforming growth factor-β (TGF-β) plays a critical role in the development, homeostasis, and repair of the cartilage. TGF-β sub-pathway have a protective function in articular cartilage, but the expected role is altered in human OA cartilage because the expression levels of TGF-β isoforms are negatively correlated with the expressions of main proteins in human cartilage, i.e., type II collagen and aggrecan [68]. The results are consistent with a strong correlation between expressions of TGFβ1 and MMP-13 in OA-affected cartilage that TGF-β can upregulate the levels of MMP13 primarily through the SMAD independent pathway, suggesting TGF-β switches from a protective role observed from in vitro studies to a negative factor in OA-affected cartilage [69]. Insulin-like growth factor-1 (IGF-1) is another biomarker associated with the production of cartilage matrix proteins and cell proliferation [70]. IGF-1 could induce enhancement of type II collagen and reduction in MMP-13 in rat endplate chondrocytes, but this regulation is through different signaling pathways. The PI3K pathway mainly transduces the IGF-1 effect on type II collagen expression, while the extracellular signal-regulated kinase (ERK) pathway mediates the IGF-1 inhibitory effect on MMP-13 expression [71].

### 3.4. Proteases

SIRT1 (Sirtuin 1) is a nicotinamide adenosine dinucleotide (NAD)-dependent deacetylase that removes acetyl groups from various proteins. Its activity is negatively correlated with increased expression of MMP-13 mediated by an intermediate factor LEF1 in human primary chondrocytes [72]. This effect was confirmed in the SIRT1 knockout mouse models of OA that SIRT1 repressed LEF1 protein expression, reducing LEF1 transcriptional activity, sequentially lifting its inhibitory effects on downstream MMP-13 expression in the articular cartilage [73]. Another study further exhibited that increased SIRT1 prevents apoptosis and ECM degradation in resveratrol-treated OA chondrocytes via the Wnt/β-catenin signaling pathways [74]. At the same time, LEF1 is a key mediator of the Wnt/β-catenin signaling pathway, which interacts with β-catenin to regulate Wnt target gene expression. MMP-13 is a target protein downstream of the Wnt/β-catenin signaling pathway [73]. These results suggest SIRT1 may downregulate MMP-13 in OA via Wnt/β-Catenin/LEF1 pathway. High-temperature requirement A1 (HTRA1), a serine protease, is strongly expressed in both human OA cartilage and the articular cartilage in mouse models of OA [75]. Once HTRA1 degrades the pericellular matrix components, including fibronectin, matrilin3, collagen oligomeric matrix protein, biglycan, fibromodulin, and type VI collagen, the membrane of the chondrocyte is exposed to the type II collagen, activating the transmembrane discoidin domain-containing receptor 2 (DDR2), a cell surface collagen receptor. DDR2 induces the upregulation of MMP-13 in response to its cartilage-specific ligand, type II collagen, which results in further degradation of the interterritorial matrix [76].

### 3.5. Receptors

Low-density lipoprotein (LDL) receptor-related protein 1 (LRP1) is a type I transmembrane cell surface receptor that can internalize extracellular proteins. It has been demonstrated that LRP1 is the major endocytic receptor of MMP-13 in human chondrocytes through directly binding to MMP-13 via hemopexin domain, mediating its internalization for subsequent lysosomal degradation [77]. This was supported by experiments in which the addition of receptor-associated protein (a ligand-binding antagonist for the LDL receptor family) or gene silencing of LRP1 markedly inhibited the cellular uptake of proMMP-13 from culture media. Osteoclast-associated receptor (OSCAR), an immunoglobulin-like collagen-recognition receptor, was reported increased during OA pathogenesis in human and mouse articular cartilages [78]. It was demonstrated that the inhibition of OSCAR activity by OSCAR deletion or treatments with human OSCAR-Fc fusion protein attenuates OA development. OSCAR deficiency resulted in downregulated expression of the ECM-degrading enzymes, such as MMP-3, MMP-13, and ADAMTS5 (a disintegrin and metalloproteinase with thrombospondin motifs 5), and upregulation of aggrecan and type II collagen. These results collectively suggest that OSCAR is involved in OA pathogenesis in mice and indirectly associated with the expression of MMP-13. Integrins are cell surface receptors that can bind cartilage ECM proteins to regulate cell proliferation, differentiation, and matrix remodeling [79]. For instance, the matrix protein fibronectin fragment (FN-f) stimulates chondrocytes to produce MMP-13 through binding with α5β1 integrins [80]. An increase in the specific MMP-cleavage of collagen type II is observed with age, accompanied by a corresponding upregulation of MMP-13 due to the increased anaplastic lymphoma kinase (ALK) ratio of ALK1/ALK5 with age [81]. In chondrocytes from aged and OA cartilage, the ratio of TGF-β receptor ALK1/ALK5 increases, leading to downregulation of the TGF-β pathway and shift from matrix synthesis activity to catabolic MMP expression [82]. These results indicate that the partial reason for enhanced collagen type II degradation by MMP-13 may be attributed to the increased ratio of ALK1/ALK5 with age. The sex hormone estrogen plays a critical role in OA pathogenesis in women over 50 years old or after menopause due to reduced estrogen levels. Estrogen receptor (ER) expressed in joint tissue and chondrocytes directly regulates some target genes’ transcripts via DNA binding. MMP-13 mRNA levels are significantly suppressed by 17-β-estradiol (E2) in the articular chondrocytes of female patients, indicating that estrogen acts via ER to inhibit the catabolic activity of MMP-13 [83].

### 3.6. Others

Leptin, a peptide hormone involved in maintaining insulin sensitivity and contributing to the sensation of satiety, is expressed at very high levels in obese individuals. It appears to be correlated to with OA by intervening the level of MMP-13 expression. Down-regulation of leptin mRNA translation via small interference RNA (siRNA) inhibits MMP-13 expression in cultured osteoarthritic chondrocytes [84].

Many other proteins, like adiponectin [85], nuclear protein-1 [86], and estrogen [87], have also been found to be amplified accompanied with increased expression of MMP-13 in OA chondrocytes. It should be noted that the mediators of MMP-13 are not limited to those mentioned above.

## 4. Epigenetic Regulation of MMP-13

The “NIH Roadmap Epigenomics Project” defined epigenetics as both heritable changes in gene activity and expression, and stable, long-term alterations in a cell’s transcriptional potential that are not necessarily heritable [88]. The epigenetic regulation will not change the underlying DNA sequence, which is contrary to gene regulation by changes in the DNA sequence. Thus, epigenetic mechanisms regulate gene expression either by affecting gene transcription or by acting post-transcriptionally [89]. Although OA’s specific mechanism is unclear, genetic changes are considered critical factors in its pathology. There is increasing evidence that gene expression can be regulated by epigenetic processes, including DNA methylation, histone modification, and non-coding RNAs (Figure 3), as outlined in the following sub-chapters.

### 4.1. DNA Methylation

DNA methylation involves adding a methyl group to the 5′ position of cytosine within a CpG dinucleotide to form 5-methylcytosine in the presence of DNA methyltransferase (DNMT) without changing the DNA sequence [90]. DNA methylation is associated with transcriptional repression and is accomplished by blocking the binding of transcription factors to gene promoters and by altering the chromatin structure through the recruitment of repressive chromatin remodeling complexes [91]. The demethylation of specific CpG dinucleotides within MMP-13 promoters has been shown to alter transcription factor binding and thereby increase MMP-13 transcription in OA cartilage [92].

DNA methylation involved in the MMP-13-driven OA process can directly target the MMP-13 promoter. For example, Roach et al. [93] first investigated the abnormal gene expression of clonal OA chondrocytes is associated with heritable epigenetic alterations in the DNA methylation pattern. They found the overall percentage of nonmethylated sites for MMP-13 were significantly increased in OA patients (20%) compared with controls (4%), but not all CpG sites were equally susceptible to demethylation. The authors identified that both the −134 and −110 sites in the MMP-13 promoter became demethylated during the OA process, even at the early stage. Another study further showed that methylation of the −110 bp CpG site, which resides within a hypoxia-inducible factor (HIF) consensus motif in the MMP-13 promoter, produced the most significant suppression of its transcriptional activities. Methylation of the −110 bp CpG site in the MMP-13 promoter inhibited its HIF-2α-driven transactivation and decreased HIF-2α binding to the MMP-13 proximal promoter by chromatin immunoprecipitation assays [92]. DNA methylation can also target the promoters of genes encoding MMP-13-mediated proteins, further activate the downstream signaling pathway, and eventually lead to chondrocyte hypertrophy and cartilage destruction [94]. RUNX2 promoter activity is increased by demethylation of specific CpG sites in the P1 promoter. Overexpression of RUNX2 significantly enhances MMP-13 promoter activity, independent of the MMP-13 promoter methylation status, which may result in high expression of its protein product and further promote the transcriptional activity of MMP-13 in OA [95].

### 4.2. Histone Modification

Histones are basic proteins that associate with DNA in the nucleus and help condense it into chromatin, which is composed of DNA-wrapped protein octamers. Histone modification can alter the chromatin conformation and influence the binding of transcription factors with the promoter region [96]. Acetylation/deacetylation and methylation/demethylation of histones are the primary modifications studied in OA [97]. Acetylation decreases the binding ability of histones with DNA, allowing the transcription factor’s binding leading to the initiation of gene expression. On the contrary, deacetylation carried out by histone deacetylases (HDAC) encourages high-affinity binding between the DNA backbone and histones, resulting in preventing the binding of transcription factors [98].

HDAC expression promotes chondrocytes’ catabolic activity, and several HDACs are upregulated in OA chondrocytes, including HDAC1, HDAC2, and HDAC7 [99]. Overexpression of HDAC1 and HDAC2 represses transcription of ECM genes, including ACAN and COL2A1, whereas HDAC7 induces transcription of the matrix-degrading enzyme MMP-13. Higashiyama et al. found that the enhanced HDAC7 promotes cartilage destruction in OA patients by inducing the expression of MMP-13. The knockdown of HDAC7 by siRNA in SW 1353 human chondrosarcoma cells induced by IL-1β leads to a decrease in the expression of MMP-13 [100]. In addition, HDAC3 has also been shown to inhibit the phosphorylation of extracellular ERK and the downstream target gene activity in chondrocytes [101]. Ablation of HDAC3 in chondrocytes increased the temporal and spatial activation of Erk1/2 by decreasing the dual-specific phosphatase 6 (Dusp6), resulting in increased Runx2 phosphorylation and MMP-13 activation as downstream effects of activated Erk pathway. It is likely that HDAC3 deletion is directly affecting Runx2 activity and histone acetylation of the MMP-13 gene, in addition to controlling Erk1/2 activity through Dusp6.

### 4.3. Non-Coding RNAs

Non-coding RNAs (ncRNA) are functional RNA molecules that produce transcripts functioning as structural, catalytic, or regulatory RNAs rather than being translated into proteins. Based on the length, ncRNAs are classified as short ncRNAs (<30 nucleotides) and long ncRNAs (lncRNAs, >200 nucleotides) [102]. Further, short ncRNAs mainly include three types-microRNAs (miRNAs), short interfering RNAs (siRNAs), and P-Element induced wimpy testis (PIWI)-interacting RNAs (piRNAs) [103]. These ncRNAs regulate gene expression at transcription, splicing, or translation levels. Recent advances in ncRNAs have revealed their importance in the pathogenesis of OA [104].

#### 4.3.1. Micro RNA

miRNAs are endogenous, single-stranded non-coding RNA in cytoplasm, usually 20–23 base pairs in length. They are involved in the posttranscriptional regulation of gene expression through binding to target mRNAs via complementary base pairing between the miRNA and the “seed sequence” present in the 3′-untranslated region (3′-UTR) or the open reading frame (ORF) of the target mRNA [105]. The incomplete base pairing can regulate gene expression by translational suppression, mRNA cleavage, and deadenylation [106,107].

Previous studies have shown that more than 30 miRNAs expressed in human joint tissue are related to cartilage homeostasis and OA development [108]. Among the miRNAs, some direct negative regulators for the expression of MMP-13 have been investigated, including miR-9, miR-146a, miR-127-5p, miR-27b, miR-320, miR-411, and miR-148, which have a direct binding site in the 3′-untranslated region (3′-UTR) of MMP-13 mRNA. The miR-9 expression is repressed while the MMP-13 expression level is elevated in OA cartilage tissues compared with normal specimens, in which miR-9 inhibits the expression level of MMP-13, thus suppressing its inhibitory effects on COL2A1 and enhancing COL2A1 expression levels, which consequently antagonizes the pathogenesis of OA. [109]. The results are consistent with previous studies conducted by Gu et al. [110] and Song et al. [111]. The miR-146 expression is correlated to cartilage degradation measured by Mankin scale in OA patients. miR-146a is significantly higher at grade I OA and lower at grade II and III OA. Furthermore, the variation in the expression of miR-146a is inversely related to the expression level of MMP-13 at a similar cartilage grade, but the increasing cartilage degradation is in parallel with increased MMP13 mRNA. Therefore, the expression of miR-146a is significantly higher during the early stages of OA than during the later stages [112]. The miR-320, which is expressed during the late stages of chondrogenic differentiation, was also significantly downregulated in OA cartilage. miR-320 targets MMP-13 during chondrogenesis and in IL-1β-activated chondrocytes [113]. miR-127-5p is an important regulator of MMP-13 in human chondrocytes and may contribute to the development of OA [114]. Upregulation of MMP-13 expression by IL-1β was correlated with downregulation of miR-127-5p expression in human chondrocytes. miR-127-5p suppressed IL-1β-induced MMP-13 production and the activity of a reporter construct containing the 3′-UTR of human MMP-13 mRNA. In contrast, treatment with anti-miR-127-5p remarkably increased reporter activity and MMP-13 production. Therefore, miR-127-5p could function as a direct negative regulator of MMP-13 in the development of OA. Overexpression of miR-27b also suppressed the activity of a reporter construct containing the 3′-UTR of human MMP-13 mRNA and inhibited the IL-1β–induced expression of MMP-13 protein in chondrocytes [115]. MMP-13 has also been identified as a direct target gene of miR-411 in chondrocytes. Overexpression of miR-411 inhibited the MMP-13 expression and increased the expression of type II collagen and type IV collagen expression in chondrocytes, suggesting that miR-411 is a crucial regulator of MMP-13 in chondrocytes and may respond to the development of OA [116]. Similarly, miR-148a is downregulated in OA cartilage, while the overexpression of miR-148a increases ECM ingredients accompanied by decreased production of degrading enzymes, including MMP-13 in chondrocytes [117].

Moreover, several miRNAs that are downregulated in OA indirectly enhance the MMP-13 expression, including miR-27a, miR-29a, miR-140, miR-222, miR-488, miR-602, miR-608, miR-24, miR-148a, miR-222, and miR-125b-5p. It was first reported that both miR-27a and miR-140, as possible regulators of MMP-13 and IGFBP-5, are reduced in OA chondrocytes. miR-140 could act directly on decreasing IGFBP-5 expression, but miR-27a indirectly decreases both MMP-13 and IGFBP-5 [118]. Co-transfection of miR-29a and miR-140, which are significantly downregulated in OA, notably affected the MMP-13 and TIMP1 gene expression that regulates ECM. Although co-transfection of miR-29a and miR-140 did not show a synergistic effect on MMP-13 protein expression and type II collagen release, both of them can significantly suppress the protein abundance of MMP-13 and restore the type II collagen release in IL-1β treated chondrocytes. The mediating effect on ECM caused by miR-29a and miR-140 may be explained by the restoration of TIMP1 that is able to promote cell proliferation and has an anti-apoptotic function [119]. miR-222 was significantly downregulated in OA chondrocytes, and its overexpression suppressed apoptotic death by downregulating HDAC-4 and MMP-13 levels; the treatment of chondrocytes with the HDAC inhibitor suppressed MMP-13 protein level and apoptosis. Altogether, miR-222 regulates MMP-13 via targeting HDAC-4 in the pathogenesis OA [120]. miR-488, which is also downregulated in OA chondrocytes, plays a protective role in OA pathogenesis by decreasing cartilage degradation by targeting the zinc transporter SLC39A8/ZIP8) since Zn^2+^ is required for the catalytic activity of MMP-13 [121]. Expression of sonic hedgehog (SHH) was inversely correlated with the expression of miR-608 in damaged cartilage and IL-1β-stimulated chondrocytes. Overexpression of miR-602 or miR-608 inhibited SHH mRNA expression, and stimulation with SHH-protein upregulated the MMP-13 expression in OA chondrocytes. Hence, suppression of miR-602 and miR-608 may contribute to the enhanced expression of MMP-13 in OA via SHH [122]. miR-24 is identified as a negative regulator of p16INK4a, which is a consequence of inherent age-associated disorders and sufficient to induce the production of MMP-13 expressed in OA chondrocytes, suggesting the downregulation of miR-24 is consistent with the increased production of MMP-13 [123]. Additionally, miR-125b-5p acts as a negative co-regulator of inflammatory genes, including MMP-13, via targeting TRAF6/MAPKs/NF-κB pathway in human OA chondrocytes [124].

However, some other miRNAs, like miR-33a, miR-181b, miR-145, miR-16-5p, and miR-483, are positively correlated with the expression of MMP-13 in OA chondrocytes. The upregulation of these miRNAs indirectly leads to an increased level of MMP-13. For example, treatment of normal chondrocytes with miR-33a resulted in significantly reduced ABCA1 (ATP-binding cassette transporter A1) and ApoA1 (apolipoprotein A1) expression levels, which were accompanied by elevated levels of MMP-13, promoting the OA phenotype, but the effects were reversed by miR-33a inhibitor [125]. Similarly, the use of an inhibitor to alter miR-181b levels can reduce MMP-13 expression, while inducing type II collagen expression [126]. Overexpression of miR-145 in OA chondrocytes induced by IL-1β resulted in a significant downregulation of ACAN and COl2A1, and increased expression of MMP-13. This effect of miR-145 on impaired ECM in OA cartilage is through direct targeting of Smad3 protein, which is essential for chondrocyte homeostasis [127]. It is reported miR-16-5p may also facilitate catabolism in cartilaginous tissues under the influence of SMAD3 [128]. The expression of miR-483 was significantly upregulated in murine OA, which was negatively correlated with the expression of BMP-7 and TGF-β, but positively correlated with MMP-13 [129].

#### 4.3.2. Small Interfering RNAs (siRNAs)

siRNAs are artificially synthesized 19–23 nucleotide long double-stranded RNA molecules that can be used to “interfere” with the translation of target protein by binding to and promoting the degradation of messenger RNA (mRNA) at specific sequences to block its expression [130].

siRNA of MMP-13 or ADAMTS5 has been tested in surgically induced mice OA model to evaluate their effects by the individual or combined intra-cellular injection compared with a control group (non-targeting siRNA). Significant improvement was observed in all three siRNA-treated groups compared to the control siRNA-injected group. The degree of OA progression of the combined group was less than that of the ADAMTS5 siRNA-only group, whereas the combined injection of MMP-13 and ADAMTS5 siRNA resulted in almost the same inhibitory effects as MMP-13 siRNA alone on cartilage degradation at the early phase of OA [131]. The inhibitory effect of the MMP-13 siRNA on OA is consistent with the results previously studied [132,133].

#### 4.3.3. Long Non-Coding RNAs (lncRNAs)

lncRNAs are fundamental regulators of transcription, and function as a signal, decoy, scaffold, guide, enhancer RNAs, and short peptides to regulate transcription in response to various stimuli [134]. Many lncRNAs have been identified differentially expressed in OA patients vs. Normal [135]. Recent literature showed that several lncRNAs potentially play a role in OA associated with MMP-13.

A lncRNA, termed as cartilage injury-related lncRNA (lncRNA-CIR), was highly expressed in OA cartilage. In contrast, silencing of lncRNA-CIR was confirmed to promote the formation of collagen and aggrecan and reduce the expression of matrix-degrading enzymes, such as MMP-13 and ADAMTS5 [136]. Another long non-coding RNA, GAS5, plays a critical role in the regulation of miR-21 in OA [137]. GAS5 was identified as a direct target of miR-21, and the overexpression of GAS5 was subsequently found to promote OA pathogenesis by increasing MMP-13 expression levels [137]. The expression of lncRNA-UCA1 (urothelial cancer associated 1) was also found upregulated in the OA cartilage, and it is negatively correlated with the expression of miR-204-5p. Moreover, MMP-13 was a direct target gene of miR-204-5p in the chondrocytes. The results indicated that LncRNA-UCA1 enhances MMP-13 expression by inhibiting miR-204-5p in human chondrocytes [138].

In addition, circular RNAs (circRNAs) belong to the family lncRNA that, unlike linear RNAs, are characterized by a covalently closed circular RNA structure lacking 5′ cap and 3′ poly-adenylated tails [139]. It has been reported that circRNAs function as miRNA ‘sponges’ that naturally sequester and competitively suppress miRNA activity to affect cell behavior [140]. In a study to identify circRNA expression and explore the function of chondrocyte extracellular matrix related circRNAs (circRNA-CER) in cartilage, five miRNA binding sites for circRNA-CER are identified, including miR-636, miR-665, miR-217, miR-646, and miR-136, which can match the sequence of the circRNA-CER 3′UTR. miR-136 fits the 3′UTR of MMP-13 to suppress its expression, and knockdown of circRNA-CER by siRNA (si-CRE) in OA chondrocytes induced decreased MMP-13 and increased COLOA1 and AGGRN. However, as a consequence of co-transfection of the miR-136 inhibitor and si-CER into chondrocytes, the repression of MMP-13 via circRNA-CER knockdown was reversed by the miR-136 inhibitor, and the effect regarding the high expression of COL2 and ACAN was also eliminated. The results demonstrated that circRNA-CER regulated MMP-13 expression by functioning as a competing endogenous RNA and participated in the process of chondrocyte ECM degradation [141].

## 5. Selective Inhibitors of MMP-13

Since MMP-13 plays a crucial role in OA, it has become a hot spot that researchers are paying attention to. We might be able to treat OA if we are able to detect or to synthesize a species that is able to block a specific step in the transcriptional regulation pathway of MMP-13, to inhibit its synthesis, or to combine with MMP-13 to inhibit its activity. Such substances are called MMP-13 inhibitors. For example, there are some natural compounds such as resveratrol [142], curcumin [143], epigallocatechin-3-gallate [144], showing protective effects against matrix degradation and inflammation in OA-affected chondrocytes by indirectly inhibiting the expression of MMP-13. However, these natural compounds still have potentially toxic effects on other human tissues because they do not work only to MMP-13. Here we introduce some selective MMP-13 inhibitors, which means the compounds bind exclusively to MMP-13 without acting on other substrates. They can be divided into two types: biologically synthesized inhibitors and chemically synthesized inhibitors.

### 5.1. Chemically Synthetic Inhibitors

MMPs have a conserved active site motif, where a tris(histidine)-bound zinc ion acts as the catalytic site for substrate hydrolysis. MMP inhibition can be achieved by chelating the active site zinc ion via Zn^2+^ binding groups (ZBGs), including hydroxamic acid, carboxylates, thiols, and phosphorous acid-based ligand. The hydroxamic acid functionality has traditionally been the ligand of choice for the most potent MMP inhibitors. A number of different hydroxamic acid-based MMP inhibitors have been reported showing efficacy in the mono-iodoacetate-induced model of joint degeneration in rats [145]. However, the clinical utility of broad-spectrum MMP inhibitors has been restricted by developing musculoskeletal syndrome (MSS) due to high structural similarity among the various MMPs [146].

#### 5.1.1. Zinc-Binding Inhibitors

To avoid broad MMP inhibition owing to the high structural homology of the different MMPs and to reduce the off-target effects observed in clinical trials, researchers have developed MMP-13 inhibitors with superior selectivity profiles over traditional MMP inhibitors through binding deeply in the S1′ region of MMP-13 [147]. This kind of zinc-binding inhibitors generally contain not only a ZBG to chelate with catalytic Zn^2+^, but also a P1′ fragment that can be accommodated in the S1′ subsite of the enzyme active site.

A series N-O-Isopropyl sulfonamido-based hydroxamates, in which different aryl substituents on the sulfonamidic portion are introduced, are synthesized and evaluated as MMP-13 inhibitors for potential therapeutic agents of OA [148]. Among these inhibitors, compound 5 exhibited a nanomolar inhibiting activity for MMP-13 (IC_50_ = 3.0 ± 0.2 nM) and was highly selective for this enzyme compared to other MMPs. This compound acted as a slow-binding inhibitor of MMP-13 and was demonstrated to be effective in an in vitro collagen assay and a cartilage degradation model. A series of carboxylic acid inhibitors of MMP-13 were also designed for the treatment of OA without significantly inhibiting the related MMP-1 or TNF-α converting enzyme (TACE) [149]. A compound 24f, optimized from carboxylic acid lead 9, was identified as a subnanomolar inhibitor of MMP-13 (IC_50_ value 0.5 nM and Ki of 0.19 nM) having no activity against MMP-1 or TACE (IC50 of >10,000 nM), and significantly reduced proteoglycan release following oral dosing at 30 mg/kg (75% inhibition) and 10 mg/kg (40% inhibition) in a rat model of MMP-13-induced cartilage degradation [149]. Recently, it has reported the discovery of series of pyrimidine-2-carboxamide-4-one-based MMP-13 zinc-binding inhibitors as exemplified by compound 35, which is characterized by having a triazolone as the zinc-binding moiety connected via a biphenyl spacer with a sulfonyl linkage to the P1′ group, quinazoline-2-carboxamide. The triazolone inhibitor 35 exhibited excellent potency (IC50 = 0.071 nM) and selectivity (greater than 170-fold) over other MMPs (MMP-1, 2, 3, 7, 8, 9, 10, 12, and 14), but slow oral availability in rat pharmacokinetic studies [150]. A novel series of fused pyrimidine compounds that possess a 1,2,4-triazol-3-yl group as a ZBG were developed. Among them, 31f exhibited excellent potency for MMP-13 (IC50 = 0.036 nM) and selectivities (greater than 1500-fold) over other MMPs (MMP-1, -2, -3, -7, -8, -9, -10, and -14) and was shown to protect bovine nasal cartilage explants against degradation induced by interleukin-1 and oncostatin M [151].

#### 5.1.2. Non-Zinc Binding Inhibitors

Another class of MMP-13 inhibitors were produced not to bind to the catalytic zinc ion, but to burry themselves deeper in the S1′ pocket, occupying the specificity loop leading to inhibitors that combine high potency with appealing selectivity profiles. An advantage of non-zinc-binding MMP inhibitors is a potential reduction in non-specific, off-target metalloenzyme inhibition. A number of highly selective and potent MMP-13 inhibitors with a variety of structural scaffolds, including diphenyl ethers, biaryls (aryltetrazoliums, arylfurans, pyrazole-indoles), pyrimidines, and aryl/cycloalkyl-fused pyrimidines, have been explored [152].

Several groups with various structural classes have shown efficacious results and, at the same time, can overcome safety problems associated with MMP inhibitors developed so far for the potential treatment of OA. For example, some pyrido [3,4-d] pyrimidin-4-ones were discovered as orally active and specific MMP-13 inhibitors for the treatment of OA. Some derivatives, such as compound 10a, possessed favorable absorption, distribution, metabolism, and elimination and safety profiles. More significantly, 10a effectively prevented cartilage damage in rabbit animal models of OA without inducing musculoskeletal side effects when given at extremely high doses to rats [153]. A group of highly selective and orally bioavailable MMP-13 inhibitors has been identified based upon a (pyridin-4-yl)-2H-tetrazole scaffold. The desired compound 29b demonstrated potent inhibition of full length MMP-13 (K_i_ = 4.4 nM) and excellent oral bioavailability in the rat (F = 100%, 1 mg/kg dose) with low clearance (14 mL/min/kg, T1/2Eff = 2.8 h). It was also found to inhibit production of type II collagen neoepitope, a biomarker of cartilage degradation, and afforded cartilage protection in a preclinical rat OA model [154]. A non-zinc binding MMP-13 selective inhibitor (4-methyl-1-(S)-({5-[(3-oxo-3,4-dihydro-2H-benzo[1,4]oxazin-6-ylmethyl)carbamoyl]pyrazolo[1,5-a]pyrimidine-7-carbonyl}amino)indan-5-carboxylic acid) is distinguished for its remarkable durability and minimized systemic exposure by IA injection in knee joint of rats. It is able to attain high, sustained concentrations (≥2 μM for 8 weeks) and sustained efficacy (100% inhibition for 3 weeks) without gross changes in the joint or signs of MSS [155]. PF152 was shown to decrease human cartilage degradation ex vivo and reduce cartilage lesions with decreased type II collagen and aggrecan degradation in dogs with OA induced by partial medial meniscectomy [156]. However, additional preclinical testing of PF152 indicated significant nephrotoxicity due to the compound’s accumulation in the kidneys mediated by human organic anion transporter 3. Thus, a follow-up analysis produced an optimized compound N-(4-Fluoro-3-methoxybenzyl)-6-(2-(((2S,5R)-5-(hydroxymethyl)-1,4-dioxan-2-yl)methyl)-2H-tetrazol-5-yl)-2-methylpyrimidine-4-carboxamide lacking the previously observed preclinical toxicology at comparable exposures [157].

Although all of these compounds showed very impressive selectivity profiles toward MMP-13, no such molecules have yet been approved for use in the clinic, as some concerns remain about poor solubility, permeability, biodistribution, metabolic stability, or bioavailability, and thus the search for new MMP-13 inhibitors continues [150].

### 5.2. Biologically Synthetic Inhibitor

Monoclonal antibody (mAb) therapy is one of the biological treatments aiming to slow structural progression, control inflammation, and relieve the pain of OA. Several kinds of mAbs, including anti-ADAMTS mAbs, anti-IL-1 mAbs, anti-NGF mAbs, and anti-VEGF mAbs, have been developed for the treatment of OA in experimental to clinical studies and show the potential to be disease-modifying anti-OA drugs (DMOADs) [158]. Due to the pivotal role of MMP-13 in the pathogenesis of OA, anti-MMP-13 mAbs is also a targeted therapeutic approach. An interesting study by Naito et al. demonstrated the development of a novel MMP-13 neutralizing antibody by immunization with a synthetic peptide, which selectively binds the active MMP-13 and is highly selective over other MMPs [159]. Despite the encouraging results using antibodies, the potency is also limited by the immunogenicity of functional sites, rapid evolvement of genetic alteration occurring at protein surfaces under pathological conditions, limited administration routes, difficulties of penetrating cartilage, and expense [158,160]. Therefore, the therapeutic potential of antibodies against MMM-13 for OA has yet to be realized.

So far, there are no clinical trials for both chemically and biologically synthetic inhibitors selective for MMP-13. These inhibitors are still under development to eliminate side effects that cause great harm. Hence, more research is needed to evaluate their safety and efficacy before administration to patients.

## 6. Conclusions

In this review, we discuss the recent advances made in understanding the role of MMP-13 in OA development and the therapeutic potential of MMP-13 inhibition in that condition. MMP-13 is regulated by multiple pathways, and some of the regulatory mechanisms are mutually causal. Even so, MMP-13 plays a pivotal role in cartilage destruction for. Therefore, elucidation of the biological processes provides a foundation with which to understand mechanisms of MMP-13 regulation in OA and potentially to refine the specificity of MMP-13 inhibition as a therapeutic strategy for OA. However, limited data are available on the role of MMP-13 inhibitors in the treatment of OA, and further research is needed to develop highly selective agents to avoid the side effects of non-selective MMP inhibitors. In the future, MMP-13 inhibitors may bring a breakthrough in the treatment of OA.

## Data Availability

Not applicable.

## Abbreviations

ADAMTS5: a disintegrin and metalloproteinase with thrombospondin motifs 5; ALK: anaplastic lymphoma kinase; AP-1: activator protein 1. 3′-UTR: 3′-untranslated region; C/EBPb: CCAAT/enhancer-binding protein-b; cirRNAs: circular RNAs; circRNA-CER: chondrocyte extracellular matrix related circRNAs; COL2A1: Collagen Type II Alpha 1 Chain; DDR2: discoidin domain-containing receptor 2; DNMT: DNA methyltransferase; DMM: destabilization of the medial meniscus; Dusp6: dual-specific phosphatase 6, ECM: extracellular matrix; ELF3: E-74 like factor 3; ER: Estrogen receptor; FN-f: fibronectin fragment; LEF1: lymphoid enhancer-binding factor 1; ERK: extracellular signal-regulated kinase; HDAC: histone deacetylase; Hes1: hairy and enhancer of split-1; HIF-2α: hypoxia-inducible factor-2 alpha; IL-1β: interleukin-1 beta; IGF-1: Insulin growth factor-1; LDL: Low-density lipoprotein; LRP1: Low-density lipoprotein receptor-related protein 1; lncRNA-CIR: cartilage injury-related lncRNA; mAb: monoclonal antibody; MAPK: mitogen-activated protein kinase; miRNA: microRNA; MMP: matrix metalloproteinase; ncRNA: non-coding RNA; NF-κB: Nuclear factor kappa-light-chain-enhancer of activated B cells; NSAIDs: non-steroidal anti-inflammatory drugs; OA: osteoarthritis; ORF: open reading frame; OSCAR: Osteoclast-associated receptor; PBX3: pre-B-cell leukemia transcription factor; PI3K/AKT: phosphoinositide 3-kinase/protein kinase B; PTGS: post-transcriptional gene silencing; RUNX2: runt-related transcription factor 2; SHH: sonic hedgehog siRNAs: short interfering RNA; TACE: TNF-α converting enzyme; TIMP: tissue inhibitors of metalloproteinases; TNF-α: tumor necrosis factor-α; UCA1: urothelial cancer associated 1; ZBG: Zn^2+^ binding group.
